# Vulnerability and Resilience of Urban Traffic to Precipitation in China

**DOI:** 10.3390/ijerph182312342

**Published:** 2021-11-24

**Authors:** Min Zhang, Yufu Liu, Yixiong Xiao, Wenqi Sun, Chen Zhang, Yong Wang, Yuqi Bai

**Affiliations:** 1 Department of Earth System Science, Ministry of Education Key Laboratory for Earth System Modeling, Institute for Global Change Studies, Tsinghua University, Beijing 100084, China; zhangmin15@mails.tsinghua.edu.cn (M.Z.); liuyufu18@mails.tsinghua.edu.cn (Y.L.); xiaoyixiong@mail.tsinghua.edu.cn (Y.X.); sunwq06@163.com (W.S.); 2 Beijing Baidu Netcom Science Technology Co., Ltd., Beijing 100085, China; zhangchen_bjtu@163.com

**Keywords:** Healthy Cities, urban health assessment, urban traffic, precipitation, traffic vulnerability, traffic resilience

## Abstract

The concept of Healthy Cities, introduced by the World Health Organization, demonstrates the value of health for the whole urban system. As one of the most important components of urban systems, transportation plays an important role in Healthy Cities. Many transportation evaluation systems focus on factors such as road networks, parking spaces, transportation speed, accessibility, convenience, and commuting time, while the vulnerability and resilience of urban transportation are rarely evaluated. This study presents the preliminary progress in the evaluation of traffic vulnerability and resilience during precipitation events in 39 Chinese cities. Traffic congestion index data, derived from the Baidu Map Smart Transportation Platform, and rainfall data, derived from NASA’s global precipitation measurement, are utilized. Traffic vulnerability index, traffic resilience index, and the corresponding quantitative methods are proposed, and the analysis results are presented. This study is of value in improving the understanding of urban traffic vulnerability and resilience, and in enabling the quantitative evaluation of them in urban health assessment and the Healthy Cities program.

## 1. Introduction

The rapid advance of urbanization has brought new opportunities, as well as challenges, for urban residents [1,2,3,4,5,6]. In 1984, the World Health Organization (WHO) introduced the concept of “Healthy Cities”, with the aim of “continuously creating and promoting those physical and social environments and expanding those community resources which enable people to mutually support each other in developing all the functions of life and developing to their maximum potential” [7]. Following the concept of Healthy Cities, the Chinese government issued the Healthy China 2030 Planning Outline in 2016, which pointed out that “health is the core content of China’s sustainable development” [1,8]. In 2016, the Office of the National Patriotic Health Campaign Committee Office initiated the Healthy Cities program to carry out the healthy city assessment [9]. The Ministry of Housing and Urban–Rural Development of China launched an Urban Health Assessment program in 2019. The major aim of this program is to propose an evaluation indicator system to review the healthy status of the natural environment, built-up environment, social environment, and human beings in representative cities [10].

Transportation is one important component of urban systems. Due to the large population and active economic activities in cities, the transportation system is often under pressure, resulting in unhealthy conditions, such as traffic congestion [11,12,13,14,15]. Therefore, the healthy status of transportation systems in major cities in China has been examined in the Urban Health Assessment program [10]. Urban road networks, parking spaces, transportation speed, accessibility, convenience, and commuting time are factors that often appear in various urban traffic assessments. However, the vulnerability and resilience of urban transportation are rarely studied. By focusing on the response of urban traffic to adverse, or even severe, weather conditions [16,17,18,19,20], this study presents a useful method to quantitively evaluate the influence of precipitation on urban traffic. It not only considers the vulnerability of urban traffic during precipitation, but also pays attention to the recovery of urban traffic after precipitation. The purpose of this paper is to present the details of this study to demonstrate a feasible way to evaluate the vulnerability and resilience of urban transportation [21,22,23,24].

The rest of this paper is organized as follows: The study area and data are introduced, followed by the method of evaluating traffic vulnerability and resilience. The next section focuses on the analysis results. The advantages and limitations of this study are discussed, leading to the conclusions.

## 2. Study Area and Data Preprocessing

### 2.1. Study Area

**Figure 1:**
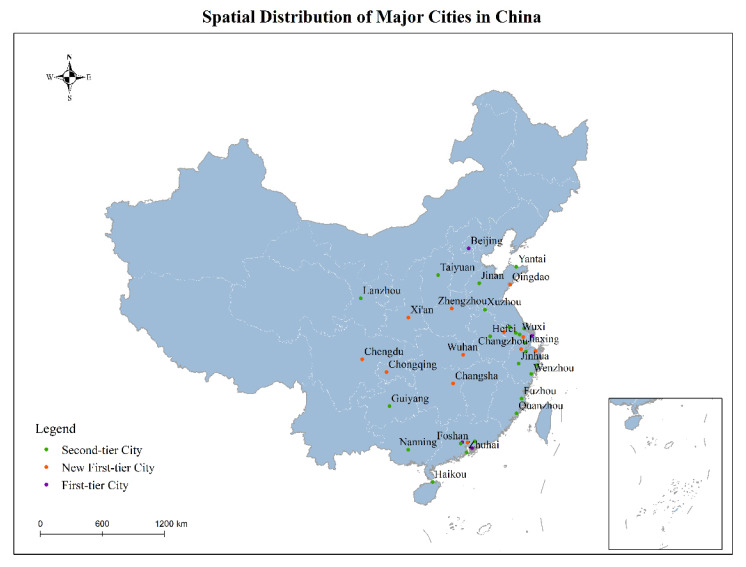
Special distribution of major cities in china.

This study selected 39 major cities in China, covering Beijing–Tianjin–Hebei urban agglomeration, Yangtze River Delta, Pearl River Delta, and several other densely populated urban agglomerations. They include four first-tier cities (Beijing, Shanghai, Guangzhou, and Shenzhen), 12 new first-tier cities (Chengdu, Suzhou, Hangzhou, Chongqing, Wuhan, Changsha, Nanjing, Zhengzhou, Dongguan, Qingdao, Ningbo, and Xi’an), and 23 second-tier cities (Wuxi, Foshan, Hefei, Fuzhou, Yantai, Jinan, Wenzhou, Quanzhou, Guiyang, Lanzhou, Nanning, Jinhua, Changzhou, Nantong, Jiaxing, Taiyuan, Xuzhou, Haikou, Huizhou, Taizhou, Zhuhai, Shaoxing, and Yangzhou). These cities are located in multiple climatic zones, from the rainy southern part of China to the less rainy northern part (Figure 1).

### 2.2. Data Preprocessing

The data used in this study mainly include urban traffic data and rainfall data. The urban traffic data are derived from the Baidu Map Smart Transportation Platform (https://jiaotong.baidu.com/, accessed on 9 January 2021), and the rainfall data are derived from NASA’s global precipitation measurement (https://gpm.nasa.gov/data/directory, accessed on 9 January 2021).

#### 2.2.1. Collection and Preprocessing of the Traffic Data

The Baidu Map Smart Transportation Platform is capable of calculating the average speed of vehicles in 100 cities in China, and the traffic congestion index in near real time. The traffic congestion index is a crucial measure of urban traffic conditions, and is obtained by dividing the actual travel time by the unobstructed travel time. A congestion index of 1.0–1.5 indicates smooth driving, with 1.5–1.8 indicating slow driving and 1.8–2.0 indicating congestion, while a congestion index greater than 2.0 indicates severe congestion [25,26].

This study uses the congestion index data of 39 cities in China over a two-year time span (from January 01, 2018, to December 31, 2019), with a time resolution of one hour. To eliminate the influence of periodic changes (working and non-working days, and rush and non-rush hours) on traffic conditions, the study focuses on the evening rush hours of working days, and selects congestion index data from 5 pm to 8 pm on weekdays in 2018 and 2019.

#### 2.2.2. Collection and Preprocessing of Rainfall Data

The global precipitation measurement provides a set of global rainfall data based on satellite observations. It has the advantages of high spatial resolution, high temporal resolution (half an hour), and wide spatial coverage. In addition, it is also highly accurate and has a strong ability to capture solid-state precipitation, heavy rainfall, and low precipitation [27].

This study adopts global rainfall data from 1 January 2018 to 31 December 2019. The latitude and longitude coordinates of the cities examined in this study are used to obtain the daily precipitation data of the grid point where each city is located. The time resolution is half an hour, with the spatial resolution being the 0.1° × 0.1° grid and the data unit being mm/hour. Two adjacent rainfall datum with the half-hour resolution are added together in order to enable the time resolution to be one hour. The time zone of the data is adjusted from UTC + 0 to UTC + 8, in order to be compatible with the traffic congestion index data.

## 3. Proposed Methods

The evening rush of the working day refers to the increase in traffic behavior caused by people coming home from get off work or participating in evening. If precipitation occurs in this period, or stops shortly before this period, its impact on urban traffic will be very significant. Thus, this research mainly focuses on this situation by proposing vulnerability index and resilience index, and performing quantitative evaluation of the vulnerability and resilience of urban traffic to precipitation in 39 cities in China. The traffic vulnerability index refers to the changes in the urban traffic congestion index under different rainfall intensities, and the traffic resilience index refers to the time window allowing urban traffic to recover to the historical congestion index after precipitation ends.

### 3.1. Traffic Vulnerability Index

The traffic vulnerability index is established to evaluate the response of different cities to different rainfall intensities, measuring urban traffic during precipitation. The basic idea of establishing this index is described as follows.

Firstly, precipitation intensity classification. Precipitation intensity refers to the amount of precipitation per unit time, which, in this study, is an hour and the unit of intensity is mm/h. The precipitation intensity, obtained based on the data from 2018 to 2019, is divided evenly by uniform interval, and the average value of urban traffic congestion index under each precipitation category is calculated as follows:

N = (max − min) ÷ interval(1)

In this equation, max and min represent the maximum and minimum hourly precipitation for the time period selected by the study, respectively, while interval means the grading interval. In this study, the interval is 0.1 mm. N is the grading number of a city.

The next step is regression analysis. Based on the results of the above classification, scatter plots are drawn and regression analysis is conducted to obtain the regression coefficient, which is also the vulnerability index of the city traffic under precipitation. This study adopts the ordinary least squares (OLS) method to estimate the regression coefficient. The hypothesis testing is performed and *p* < 0.05 is considered statistically significant.

### 3.2. Traffic Resilience Index

Traffic resilience index is established to measure the ability of the urban traffic to return to a normal state after precipitation. The basic idea of establishing this index is as follows: the traffic recovery time of the city after each precipitation event is calculated, then the average value of the traffic recovery time after all precipitation in two years (from 2018 to 2019) is obtained. The equation is as follows:

(2) $$\mathrm{Time}=\left(\sum_{i=1}^{n}\left(T_{End}-T_{Start}\right)\right)/n$$

In this equation, Time represents the time it takes for the city’s traffic to return to its normal state. $T_{Start}$ and $T_{End}$ refer to the beginning and ending moments of this process, respectively. Further, n refers to the number of precipitation events during the evening rush hours of the city in the two years (from 2018 to 2019).

$T_{Start}$ is defined as follows: $T_{RainEnd}$ is the ending moment of a precipitation event and it is also considered as the traffic recovery moment. Since this study focuses on the evening rush hours, data of the working days with precipitation during the evening rush hours and no precipitation after the rush hours are chosen. In order to eliminate the influence lag of precipitation on traffic, data of the working days with precipitation from 16:00 to 20:00 and no precipitation from 20:00 to 00:00 are chosen. The moment when precipitation ends is also the moment when the recovery process begins.

(3) $$T_{Start}=T_{RainEnd}\left(T_{RainEnd}\leq 20:00\right)$$

$T_{End}$ is defined as follows: under the influence of rainfall, the moment when the traffic congestion index reverts to the historical level is considered as the end time of the recovery process. Since the time resolution of the traffic data in the study is one hour, the traffic congestion index data of workdays without rainfall are divided at intervals of one hour. In order to eliminate the effect caused by data fluctuation, the historical level of the hourly congestion index is obtained by adding the average and the standard deviation of the congestion index together.

(4) $$\Delta I=I_{now}-\left(\mu +\sigma \right)$$

In this equation, ΔI is the change in the congestion index, I is the actual value of the congestion index, μ is the average of the historical congestion index and σ is the standard deviation of the historical congestion index. When ΔI > 0, the congestion index is higher than the historical congestion index. In this case, the urban traffic is still congested and is still recovering from precipitation. ΔI ≤ 0 means that the congestion index is equal to or lower than the historical congestion index. In this case, the urban traffic is no longer in congestion and this moment is considered as the ending moment of the recovery process.

## 4. Research Results

### 4.1. Traffic Vulnerability in 39 Cities

**Figure 2:**
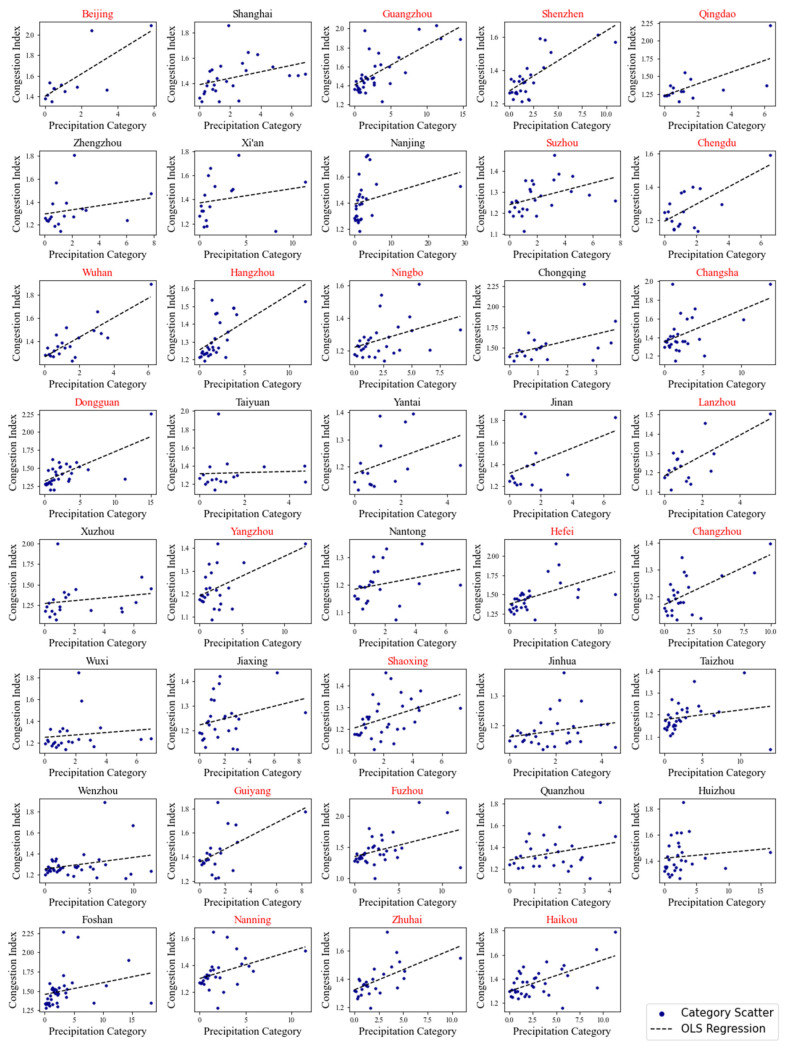
Statistical scatter plots and the ordinary least squares (OLS) regression analysis plots of the rainfall intensities and traffic congestion indexes.

To eliminate the influence of the periodic changes in traffic conditions, the study uses traffic data and precipitation data of evening rush hours (from 5 pm to 8 pm) of the working days from 1 January 2018 to 31 December 2019. Based on the traffic vulnerability evaluation method mentioned above, the traffic vulnerability index and significance test results of 39 first-tier and second-tier cities in China are obtained, and scatter plots and OLS fitting line graphs are constructed (Figure 2). The subfigures are arranged according to the level of development of each city (first-tier, new first-tier, and second-tier cities), and each tier is arranged from north to south, according to the latitude of the city. Based on the results of the regression analysis, hypothesis testing is performed. *p* < 0.05 is considered statistically significant, that is, the urban traffic is strongly affected by precipitation and the traffic vulnerability is relatively high. *p* > 0.05 is considered statistically insignificant, that is, the urban traffic is not affected by precipitation. The subfigures of the cities, where *p* < 0.05 are marked in red, are shown in Figure 2.

Following this, the k value of the traffic vulnerability index and *p* value of each city are obtained (Table 1). The results show that, for the above 39 cities, the *p* value of 21 cities is less than 0.05, and these cities pass the significance test. The *p* value of the other 18 cities is greater than 0.05. The cities that passed the significance test are Beijing, Guangzhou, Shenzhen, Qingdao, Suzhou, Chengdu, Wuhan, Hangzhou, Ningbo, Changsha, Dongguan, Lanzhou, Yangzhou, Hefei, Changzhou, Shaoxing, Guiyang, Fuzhou, Nanning, Zhuhai, and Haikou.

The traffic vulnerability index and the results of the significance test show that, among the first-tier cities, Beijing, Guangzhou, and Shenzhen pass the significance test, and Beijing has a higher vulnerability index than Shanghai and Shenzhen. Among the new first-tier cities, Qingdao, Suzhou, Chengdu, Wuhan, Hangzhou, Ningbo, Changsha, and Dongguan pass the significant test, and the vulnerability index of Qingdao, Wuhan, and Chongqing is relatively large. Among the second-tier cities, Lanzhou, Yangzhou, Hefei, Changzhou, Shaoxing, Guiyang, Fuzhou, Nanning, Zhuhai, and Haikou pass the significant test, and the vulnerability index of Lanzhou and Guiyang is relatively large.

The traffic resilience index measures the ability of the urban traffic to respond to precipitation. This index also reflects the level of urban road network construction, as well as the level of urban traffic management services. The above-mentioned cities, with a large vulnerability index, mainly have the following characteristics in terms of topography and urban traffic patterns (Table 2):

1. Rugged terrain: For example, Chongqing possesses a hilly landscape. Its terrain is rugged and the connectivity of its road network is poor. There is only a single linking road between some areas. Moreover, its population density and the density of land development is high. Another example is Guiyang. Guiyang is located in the middle of the hilly area of the Guizhou Mountains. This rugged city is crisscrossed by mountains and forests, causing difficulties in the construction of an urban road network. In addition, the city form of Guiyang is the “S” type. There are many slopes and dead-end roads in this city, and the utilization rate of the road network is relatively low.

2. Severe natural obstacles: Take Wuhan as an example. This city is divided into “Three towns” by the Yangtze River and the Han River. It is a multi-central-cluster-type city. The trunk roads crossing the river are connected by three bridges over the river, thus the traffic capacity is limited. Another instance is Lanzhou. This city’s natural geographical conditions of “two mountains facing each other, one river flowing in the middle, and the railway separating the city” make the city a valley-shaped linear city. This kind of natural bottleneck also restricts the construction of a road network system. The third case is Qingdao. As a typical bay-type city, Qingdao is located in Jiao Zhou Bay, Shandong Peninsula. Its road system is characterized by “ring-shape + radiation”, with poor connectivity, and many detours and slopes.

3. Planning bottlenecks caused by history and culture: For instance, in Beijing, due to historical and cultural factors, there are some closed yards, such as the Forbidden City and the Summer Palace. As they occupy large areas, vehicles are not allowed to pass through them, limiting the road network connectivity. In addition, the dense clusters of quadrangles and winding hutongs within the 2nd Ring Road also bring difficulties to road construction. The city features single-centered dense blocks that have formed over a long time. Thus, its traffic mainly depends on the ring road, resulting in poor density and connectivity of the road network.

The natural bottleneck of topography brings difficulties to urban planning and road network construction, resulting in low density and poor connectivity of urban road networks. According to the Road Network Density Monitoring Report of Major Cities in China, issued by the Ministry of Housing and Construction in 2020, the average road network density of major first-tier and second-tier cities in China is 6.1 km/km^2^ [28]. With the exception of Chongqing and Guiyang, the road densities of the above-mentioned cities are all lower than the average levels of their counterparts. Their road networks lack proper redundancy and are poor in substitutability. In addition, there are many slopes, detours, and dead-end roads in Chongqing, Guiyang, and Qingdao, causing poor connectivity of the road network. Therefore, the traffic in these cities is more vulnerable to precipitation.

### 4.2. Traffic Resilience in 39 Cities

This study uses traffic and precipitation data of evening rush hours of workdays from 1 January 2018 to 31 December 2019 to eliminate the impact of periodic changes in traffic conditions.

**Figure 3:**
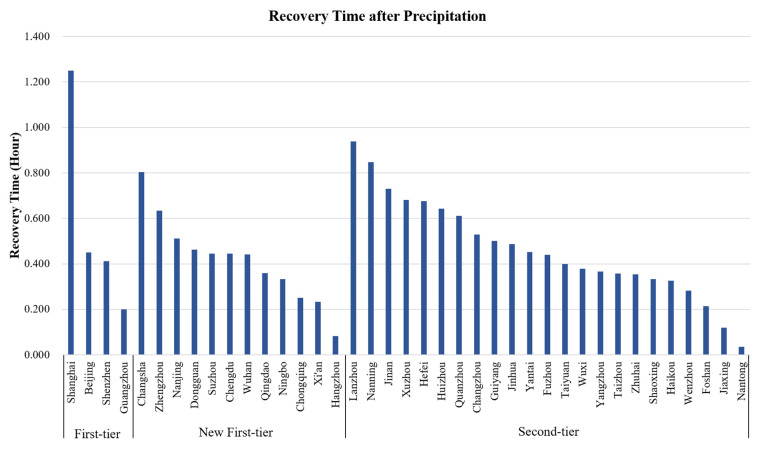
Recovery time of each city after precipitation.

The data of 39 first-tier and second-tier cities in China are analyzed, according to the above-mentioned evaluation method (Figure 3). The recovery time of each city after precipitation is calculated (Table 3).

The traffic resilience evaluation index measures the capacity of recovery and the resilience of urban traffic after precipitation. The drainage of water on the roads and the alleviation of traffic congestion after precipitation reflect the development level and management service level of the city. Based on the analysis of this index, two main factors that affect the resilience of urban traffic are concluded as follows:

(1) The size of the city: Megacities, such as Shanghai, or province capitals, such as Nanning, Jinan, Changsha, Zhengzhou, Hefei, and Lanzhou, usually cover an urban area of more than 3000 square kilometers. These large cities are weak in alleviating traffic congestion and in traffic recovery after precipitation. In contrast, cities such as Nantong, Yangzhou, Shaoxing, Wuxi, Jiaxing, and Haikou cover an urban area of less than 3000 square kilometers. They are relatively strong in alleviating traffic congestion and in traffic recovery [29].

(2) The level of economic development: Cities such as Nantong, Yangzhou, Shaoxing, Wenzhou, Wuxi, Jiaxing, and Haikou are located in the southeast coastal areas. These cities have relatively high levels of economic development, and their infrastructure is relatively complete. Although Shanghai is also highly developed in its economy, it is poor in traffic recovery after precipitation, due to its large size and population [30,31].

Cities are complex ecosystems combining nature, society, and economy. Under the impact of precipitation, it is usually small-scale developed cities that are better in traffic resilience. The lower population density of small-scale cities alleviates the pressure on easing traffic congestion. Cities that are highly economically developed are also better in traffic resilience due to their relatively complete infrastructure, dense road network, and good drainage capacity for water on the road. These factors contribute to a faster recovery of traffic to pre-precipitation levels.

### 4.3. Normalization Evaluation

**Figure 4:**
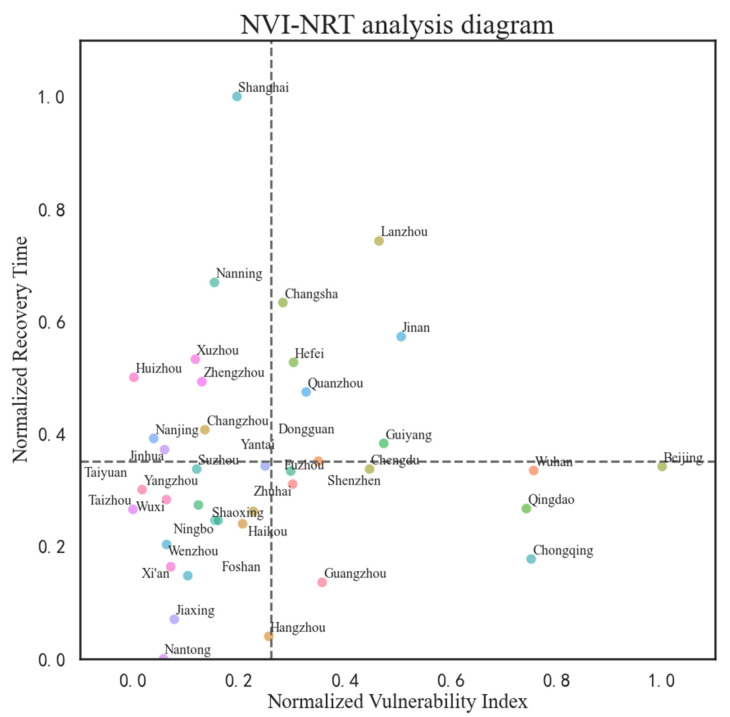
The diagram of comprehensive traffic evaluation results.

Normalization is carried out based on the traffic vulnerability index and the traffic recovery time. The vulnerability index and the recovery index are normalized to be in the range of 0–1. Let the horizontal axis be the normalized vulnerability index and the vertical axis be the normalized recovery time, and draw the NVI–NRT (normalized vulnerability index–normalized time recovery) diagram, which shows the comprehensive traffic evaluation results of the 39 cities mentioned above (Figure 4). The diagram describes the traffic resilience of cities during and after precipitation.

In the NVI–NRT diagram, the greater the abscissa, the greater the degree of vulnerability; the greater the ordinate, the longer the recovery time. The dotted line in the diagram represents the average level of the vulnerability index and the resilience index of the cities mentioned above. It is shown that cities with more complex topographical features, such as Beijing, Qingdao, Chongqing, Wuhan, Lanzhou, and Guiyang, have a high vulnerability index. Cities that are larger in size, or less economically developed, such as Shanghai, Nanning, Lanzhou, Changsha, Xuzhou, Zhengzhou, Hefei, and Jinan, have a long recovery time. The cities in the lower left corner of the diagram, such as Nantong, Jiaxing, Hangzhou, Foshan, Xi’an, Wenzhou, Ningbo, Haikou, Shaoxing, Wuxi, Yangzhou, and Taizhou, have a low vulnerability index and a short recovery time. These cities are better in traffic resilience.

## 5. Discussion

In 2019, the Central Committee of the Communist Party of China and the State Council issued the Constructing Transportation Power Outline, stating that “we should construct a modern high-quality integrated three-dimensional transportation network, enhancing the flexibility of the system. We should build a scheme preventing and controlling the damages caused by natural disasters to the transportation system, increasing its resistance to natural disasters” [22,23,24]. This study meets this demand by introducing a traffic vulnerability index, a traffic resilience index, and a quantitative method to evaluate the resilience and vulnerability of urban traffic during the precipitation events in 39 representative cities, since 2019, for the Urban Health Assessment program.

On the other hand, although the proposed indicators are useful in reflecting the vulnerability and resilience of traffic systems, they are far from a comprehensive evaluation. Enrichment of the indicators [32,33,34,35,36], proposing new quantitative methods, and performing pilot studies in other cities are highly expected.

## 6. Conclusions

In this study, a traffic vulnerability index and a traffic resilience index are proposed and utilized to assess the vulnerability and resilience of traffic affected by precipitation in 39 major cities. The results show that Beijing, Qingdao, Wuhan, Chongqing, Lanzhou, and Guiyang are very vulnerable to precipitation events, due to their relatively rugged terrain and obvious topographical obstacles, such as a large rivers or high mountains. On the other hand, Nantong, Hangzhou, Jiaxing, Foshan, Yangzhou, Shaoxing, Wuxi, and Haikou exhibit good resilience during precipitation events. The major reasons for this are that the area of these cities is not large, and the transportation infrastructure is relatively complete.

The indicators and the corresponding quantitative methods are valuable for others to refer when evaluating the vulnerability and resilience of transportation systems. Enrichment of the indicators for vulnerability and resilience, proposing new quantitative method, and performing pilot studies in other cities are expected.

## Figures and Tables

**Table 1:** k value of the traffic vulnerability index and *p* value of each city.

City	Vulnerability Index k	*p*	City	Vulnerability Index k	*p*
**Beijing**	**0.1096**	**0.0031**	Xuzhou	0.0168	0.4035
Shanghai	0.0250	0.0665	**Yangzhou**	**0.0174**	**0.0136**
**Guangzhou**	**0.0420**	**0.0000**	Nantong	0.0104	0.2566
**Shenzhen**	**0.0361**	**0.0000**	**Hefei**	**0.0363**	**0.0077**
**Qingdao**	**0.0826**	**0.0083**	**Changzhou**	**0.0186**	**0.0009**
Zhengzhou	0.0180	0.3479	Wuxi	0.0110	0.5536
Xi’an	0.0119	0.4312	Jiaxing	0.0126	0.1619
Nanjing	0.0085	0.1489	**Shaoxing**	**0.0213**	**0.0187**
**Suzhou**	**0.0170**	**0.0370**	Jinhua	0.0106	0.2019
**Chengdu**	**0.0514**	**0.0024**	Taizhou	0.0043	0.2781
**Wuhan**	**0.0840**	**0.0000**	Wenzhou	0.0110	0.1246
**Hangzhou**	**0.0314**	**0.0003**	**Guiyang**	**0.0542**	**0.0091**
**Ningbo**	**0.0207**	**0.0305**	**Fuzhou**	**0.0357**	**0.0145**
Chongqing	0.0835	0.0524	Quanzhou	0.0388	0.1404
**Changsha**	**0.0342**	**0.0039**	Huizhou	0.0045	0.5502
**Dongguan**	**0.0412**	**0.0000**	Foshan	0.0152	0.1044
Taiyuan	0.0062	0.8545	**Nanning**	**0.0206**	**0.0265**
Yantai	0.0306	0.1570	**Zhuhai**	**0.0283**	**0.0012**
Jinan	0.0577	0.1303	**Haikou**	**0.0262**	**0.0003**
**Lanzhou**	**0.0533**	**0.0024**			

Cities that pass the salience test are in bold.

**Table 2:** Characteristics of typical cities and their road network.

Name	Pattern	Form	Network Density (km/km^2^)
Beijing	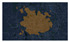	Blocks	5.7
Wuhan	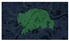	multi-central cluster	6.0
Lanzhou	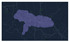	valley-shaped linear type	4.2
Chongqing	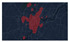	multi-central cluster	6.7
Guiyang	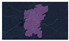	multi-central cluster, “S” type	6.2
Qingdao	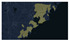	multi-central cluster	5.4

Network Density in table are quoted from road network density monitoring report of major cities in China (2020) [28].

**Table 3:** Recovery time of each city after precipitation.

City Name	Recovery Time (Hour)	City Name	Recovery Time (Hour)
Shanghai	1.250	Hefei	0.675
Beijing	0.450	Huizhou	0.643
Shenzhen	0.412	Quanzhou	0.611
Guangzhou	0.200	Changzhou	0.529
Changsha	0.804	Guiyang	0.500
Zhengzhou	0.633	Jinhua	0.486
Nanjing	0.511	Yantai	0.451
Dongguan	0.462	Fuzhou	0.440
Suzhou	0.444	Taiyuan	0.400
Chengdu	0.444	Wuxi	0.378
Wuhan	0.441	Yangzhou	0.367
Qingdao	0.359	Taizhou	0.357
Ningbo	0.333	Zhuhai	0.353
Chongqing	0.250	Shaoxing	0.333
Xi’an	0.233	Haikou	0.326
Hangzhou	0.083	Wenzhou	0.281
Lanzhou	0.938	Foshan	0.214
Nanning	0.848	Jiaxing	0.120
Jinan	0.731	Nantong	0.034
Xuzhou	0.682		

## Data Availability

The data used in this research can be found in Baidu Map Smart Transportation Platform (https://jiaotong.baidu.com/, accessed on 9 January 2021) and NASA’s Global Precipitation Measurement (https://gpm.nasa.gov/data/directory, accessed on 15 January 2021).
